# Effect of boiling on quality, microstructure and flavor of fresh peanuts

**DOI:** 10.1016/j.fochx.2026.103632

**Published:** 2026-02-03

**Authors:** Xuegang Huang, Zhenyuan Li, Gengjiu Zhao, Yumeng Hu, Fengying Gu, Qiang Wang, Qin Guo

**Affiliations:** Institute of Food Science and Technology, Chinese Academy of Agricultural Sciences/Key Laboratory of Agro-Products Processing, Ministry of Agriculture and Rural Affairs, Beijing 100193, China

**Keywords:** Fresh edible peanut, Boiling peanut, Microstructure, Flavor

## Abstract

This study systematically examines changes in the nutritional composition, microstructure, and flavor profile of fresh peanuts after boiling, identifying Jihuatian 1 as the most suitable variety due to its superior quality. Boiling significantly reduced its fat (19.73%), sucrose (42.21%), and hardness (49.11%), while increasing brittleness (59.85%) and aldehyde-type flavor compounds. These changes resulted in a distinct sweet, nutty, and floral aroma. From a microscopic perspective, boiling causes damage to the surface of peanut cells, partial rupture of cell walls and loss of cytoplasm, promoting the release of nutrients. Lipid oxidation reduces the content of unsaturated fatty acids and simultaneously generates key aldehyde flavor components. The Maillard reaction between reducing sugars and amino acids further enriches the flavor. Correlation analysis revealed key relationships between raw material properties and final product texture and flavor. The findings offer theoretical and practical guidance for optimizing quality in boiled peanut processing.

## Highlights

• Thisstudyaimstoclarifythecharacteristicchangesoffreshpeanutsbeforeandafterboiling.
• Comparedwithdifferentvarietiesoffreshpeanuts,JihuatianIhasthebestqualitycharacteristics.
• Afterboiling,thealdehyde-typeflavorsubstancesinfreshpeanutsincreasedsignificantly.
• Afterboiling,thehardnessoffreshpeanutsdecreasedsignificantlyandthecrispnessincreasedsignificantly.

## 1 Introduction

Peanuts (*Arachis hypogaea* L.) are one of the important oilseed and economic crops in the world, and are also an important source of plant protein (Wu et al., 2022). Peanut kernels contain rich fatty acids, amino acids, trace elements, and bioactive substances, which are beneficial to human health (Akram, Shafiq, & Ashraf, 2018; Zhang et al., 2022). According to USDA data, global peanut production increased year by year, reaching 49.41 million tons in 2023. Among them, 23.45 million tons were directly used for human consumption, accounting for 47.47% of the total peanut output, with huge consumption potential (Cui et al., 2023; Huang et al., 2025). Fresh peanuts, as a major category of edible peanuts, refer to peanuts harvested without drying and eaten fresh (raw peanuts) or boiled and eaten (boiled peanuts). They are loved by consumers for their delicious taste, rich nutrition, low allergy risk, etc. (Dun et al., 2019). As people's living standards continue to improve, they pay more attention to high-nutrition, high-health foods, and the market share of fresh-eaten peanuts has been increasing year by year (Bonku & Yu, 2020).

Although numerous studies have explored how peanut quality changes during processing for oil, peanut butter, and protein production (Feng, Liu, Liu, Hu, & Wang, 2014; Gong et al., 2018; Hu et al., 2019; Jiao et al., 2018; Li et al., 2022; Sithole, Ma, Qin, Liu, & Wang, 2022), research on the quality changes of fresh peanuts during boiling remains limited. Previous studies have shown that boiling reduces the allergenicity of peanuts by degrading protein epitopes (Beyer et al., 2001; Cabanillas, Jappe, & Novak, 2018; Tao et al., 2017; Wang et al., 2022). Chukwumah, Walker, Vogler, and Verghese (2007) examined the effects of various cooking methods, including boiling, on the phytochemical content of peanuts, finding that boiled peanuts contained the highest levels of total flavonoids and polyphenols, with notable increases in resveratrol and specific compounds like concutin A and genistein. Moreover, the hydrolysis of lignin during boiling generates vanillin, a key flavor compound in boiled peanuts (Sobolev, 2001). Aldehydes and limonene further contribute to the characteristic sweet and fragrant flavor of boiled peanuts. Despite these insights, no studies have yet fully characterized the microstructural changes in fresh peanuts during boiling. Existing research typically focuses on sensory and nutritional quality, but fails to address the comprehensive effects of boiling on sensory properties, nutritional content, processing quality, flavor substances, and microstructure. This gap highlights the urgent need to investigate how boiling affect the overall quality and microstructure of fresh peanuts, offering critical insights into optimizing processing techniques for improved product quality.

Some studies had shown that high-quality boiled peanuts had the characteristics of uniform appearance, bright color, delicate taste, high protein content and low fat content, which mainly focused on sensory quality indexes and nutritional quality indexes, and no in-depth studies had been conducted on their texture properties, flavor substances and microstructure. In addition, the correlation between raw material characteristics of fresh peanuts and quality indexes of boiled peanuts is not clear. The selection of high quality varieties is of great significance to improve the quality of fresh peanuts and the acceptance of consumers. In the process of boiling, different varieties of fresh peanuts raw materials have different nutrient loss due to different cell wall thickness, fat and protein content and distribution, which will directly affect the comprehensive quality of boiled peanuts. Moreover, there are no reports on the comprehensive quality and microstructure changes of different varieties of boiled peanuts.

This study investigates the effects of boiling on the quality and microstructure of fresh peanuts, using four different raw materials subjected to a standardized boiling process. The research quantitatively analyzes the nutritional content, volatile flavor components, and texture characteristics of both the raw peanuts and their boiled counterparts. By integrating sensory evaluation with microstructural analysis, the study explores how boiling influence the overall quality of fresh peanuts. Notably, this is the first systematic study to analyze the volatile flavor components of fresh peanuts, addressing consumers' growing demand for more nuanced flavor profiles in boiled peanuts. The study also establishes the relationship between the raw material characteristics of fresh peanuts and the quality indicators of boiled peanuts, offering valuable insights for selecting varieties and raw materials for optimal boiled peanut production. This work provides a crucial theoretical foundation for advancing the processing and quality control of boiled peanuts.

## 2 Materials and methods

### 2.1 Materials and reagents

The four different varieties of peanuts in this study are representative peanut varieties: Jihua 1140–1 raw peanut (IY), Jihuatian 1 raw peanut (IIY), Jihuatian 2 raw peanut (IIIY), Baisha 308 raw peanut (IVY), Jihua 1140–1 boiled peanut (IS), Jihuatian 1 boiled peanut (IIS), Jihuatian 2 boiled peanut (IIIS), Baisha 308 boiled peanut (IVS). All four varieties are adapted to the warm temperate semi-humid climate and are suitable for spring ploughing in March. The main production areas are located in Hebei Province and Shandong Province of China. The samples were provided by the Institute of Grain and Oil Crops of Hebei Academy of Agriculture and Forestry Sciences and Qingdao Agricultural University.

Reagent: Anhydrous ether, petroleum ether, ethanol, copper sulfate, potassium sulfate, sodium hydroxide, zinc acetate bought from Sinopath Group Chemical Reagent Co., LTD., sulfuric acid, boric acid, acetonitrile, glacial acetic acid, acetone bought from Shanghai Yuanye Biotechnology Co., LTD. Fluorescein isothiocyanate (FITC), bromocresol green, methylene blue and nile red were from Beijing Solaibo Biotechnology Co., LTD., and potassium ferrocyanide was from Beijing Chemical Reagent Company. phosphate buffered saline (PBS) was from Thermo Fisher Biochemical Products (Beijing) Co., LTD., and 2-octanol was from Sigma-Aldrich Company in the United States. Acetonitrile, glacial acetic acid and 2-octanol are chromatographic grade reagents, while the rest are analytical grade reagents.

### 2.2 Boiling technology

The raw materials of fresh peanuts are added to the WG-JD-33 stainless steel pot (Beijing Vogel Oriental Technology Co., Ltd.), followed by pure water at a ratio of 1:10 (*w*/w). Then the pot was covered and heated to boil, and the timer started from the boiling point of the water and continued to boil for 30 min, with the heating power set to 1200 W.

### 2.3 Sensory evaluation

After collecting expert opinions from enterprises such as Luanxian Baixin peanut planting Professional Cooperative and Shandong Jinsheng Cereals & Oils Foods Co., LTD., combined with the current standards GB/T 1532-2008, NY/T 1067-2006, NY/T 420-2017, a sensory evaluation table for fresh peanuts was developed. The sensory evaluation team was composed of ten professionally trained laboratory personnel (5 males and 5 females, ages 20–35). The color, appearance, flavor, taste and overall acceptability of the pods and kernels of the raw materials of fresh peanuts and boiled peanuts were scored and recorded according to Table 1.

**Table 1:** Sensory evaluation table of boiled peanuts.

Index			Evaluation standard		Score range
Pod	Color		Yellowish-white, glossy, unspotted Yellowish brown, dull luster, a few spots Dark brown, dull, spotty		8–10 4–7 0–3
	Appearance	Shape	Cocoon shape Normal shape, bead shape Other shapes		8–10 4–7 0–3
		Reticulum	Smooth pods, inconspicuous reticulation Pods rougher, moderately reticulated Pods are thick and rough, obvious reticulations		4–5 2–3 0–1
		Size	Length 3.0 ± 0.5 cm, width 1.5 ± 0.3 cm Length 3.0 ± 1.0 cm, width 1.5 ± 0.5 cm Remaining length and width		4–5 2–3 0–1
	Flavor		Rich taste Lighter flavor Smelly		4–5 2–3 0–1
Kernel	Color		Deep red, light red, or other bright colors Darker color Dull color		4–5 2–3 0–1
	Shape		Large and full grains, good neatness, moderate size, no cracks, no oil spots, no mold Kernels are fuller, more uniform in size, a few cracks, a few oil spots Kernels are dried out, irregularly sized, cracked, with oil spots, moldy		4–5 2–3 0–1
	Flavor		Rich taste Lighter flavor No flavor, slightly bitter, smelly		4–5 2–3 0–1
	Mouthfeel		Sweetness	Sweetness Fairly sweet Unsweetened	8–10 4–7 0–3
			Brittleness	Crisp texture Fairly brittle No brittle fracture	8–10 4–7 0–3
			Hardness	Medium hardness, chewy and soft Normal hardness, normal chewing friability Hard texture, chewing effort	8–10 4–7 0–3
			Fineness	Fine mouthfeel, small and few particles of residue after chewing Normal mouthfeel, uneven particles of residue after chewing Rough texture, more residual particles after chewing	8–10 4–7 0–3
Overall acceptability			Good Normal Poor		8–10 4–7 0–3

### 2.4 Nutritional content

The determination of fat, protein, moisture, sucrose, crude fiber, fatty acid, and amino acid contents was conducted according to the corresponding Chinese National Standards (GB 5009.6-2016, GB 5009.5-2016, GB 5009.3-2016, GB 5009.8-2016, GB/T 5515-2008, GB 5009.168-2016, and GB 5009.124-2016, respectively).

### 2.5 Texture characteristics

With reference to the texture test parameters of peanut food and nut (Li et al., 2025a), TA-TX2i texture analyzer (Stable Micro Systems, UK) was used for testing. The probe type was P/36R mm, the test speed was 2.00 mm/s, the waiting time was 0 s, and the operation mode was pressure measurement. The speed before the test is 2.00 mm/s, the speed after the test is 2.00 mm/s, the compression degree (Strain) is 40%, and the trigger stress is 15 g.

### 2.6 Qualitative and quantitative determination of flavor substances

Refer to hu's method and make modifications (Hu et al., 2019). Qualitative analysis was performed by electronic nose (German AIRSENSE company) detection technology, and quantitative analysis was performed by headspace solid phase microextraction and GC–MS technology. Electronic nose detection: the raw materials and boiled peanut samples were crushed, the air was pumped for 180 s, and the sampling was started after the baseline was stable, the determination time was 60 s, and the sampling was not hit the wall or touched, and each sample was repeated 15 times.

The sample was prepared by weighing 2 g of ground peanuts into a 25 mL headspace vial, followed by the addition of 5 μL of an internal standard solution (2-octanol, 0.819 mg/mL in methanol). HS-SPME conditions: DVB/CAR WR/PDMS SPME Arrow extraction head, balancing at 60 °C for 10 min, extraction at 60 °C for 60 min, injection port analysis for 4 min, aging at 270 °C for 2 h before the extraction head, aging at 270 °C for 15 min between samples to prevent contamination between samples. GC-Orbitrap/MS conditions: TG-5MS quartz capillary column (60 m × 0.25 mm × 0.25 μm), SSL shunt injection, shunt ratio 15:1, 250 °C, with 1.2 mL/min He carrier gas, temperature program at 40 °C for 2.5 min, rising to 70 °C at 7 °C /min. Then it rises to 120 °C at 2 °C/min for 1 min, and finally rises to 280 °C at 20 °C for 5 min, and the transmission line temperature is 250 °C. Mass spectrum conditions: resolution 60,000 FWHM (200 *m*/*z*), scanning range 35–500 m/z, full scan mode (Fullscan mode), EI mode: 280 °C, 70 eV.

OAV (Odor Activity Value) calculation: it is generally believed that substances with OAV greater than 1 contribute to the aroma of peanuts, and components with OAV greater than 10 are considered important aroma substances. The OAV calculation method is as follows:

(1) $$\mathrm{OAV}=\frac{\rho_{i}}{{\mathrm{OT}}_{i}}$$

### 2.7 Microstructure observation

Laser confocal microscope (TCS SP8, Leica, Germany) observation: The observation steps were: sample preparation → production → dyeing → machine. Different peanut samples were taken from the same part and cut into cuboids of about 2.5 mm, 1.5 mm wide and 1.5 mm high, soaked in glutaraldehyde fixing solution for 12 h, rinsed with PBS 3 times for 10 min each time, fixed with 1% osmic acid for 1.5 h, and dehydrated with 50, 60, 75, 90 and 100% ethanol solution. The peanut samples were dehydrated for 15 min each time. Finally, the peanut samples were coated with epoxy resin for semi-thin sections, and the semi-thin sections were stained with 0.2% FITC and 0.1% Nile red. Finally, the peanut microstructure was observed by CLSM (Laser confocal scanning microscope) with a magnification of 40× of the original field of view.

Scanning electron microscope (SU8010, Hitachi, Japan) observation: The observation steps were: sampling → washing → post-fixation → dehydration → drying → sticking table → gold spraying → electron microscope observation. The samples that had been fixed with glutaraldehyde were washed with phosphate buffer (PBS) for 3 h, 10 min/ time. After fixation, it was fixed with 1% osmic acid for 2 h. During dehydration, the sample was rinsed with distilled water three times for 3 min/ times, and dehydrated with alcohol gradient for 15 min/ gradient (30, 50, 60, 70, 80, 90, 95, 100). The samples were dried using the Leica CPD 030 CO_2_ critical point dryer. The ion sputter HITACHI MC1000 was used to spray the sample during gold spraying.

### 2.8 Statistical analysis

Original data recording and analysis through Excel 2016; SPSS Statistics 26 was used for data analysis (*p* < 0.05, significant difference). Origin 2022 software was used for data correlation analysis and image rendering.

## 3 Results and discussion

### 3.1 Sensory quality evaluation scores

**Fig. 1:**
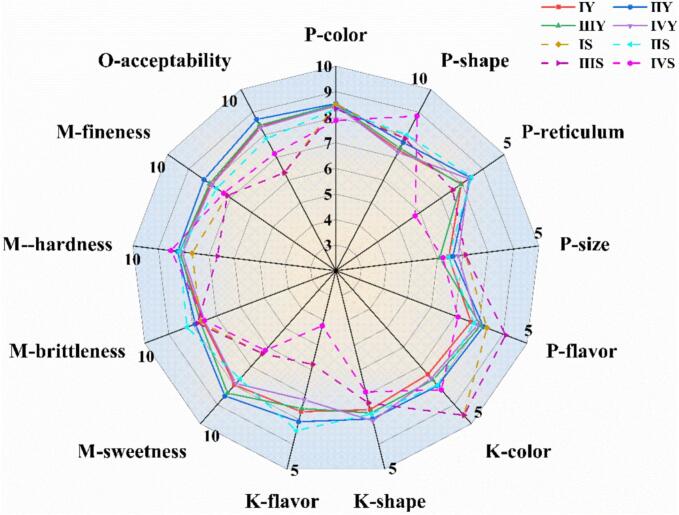
Sensory quality evaluation scores of different varieties of fresh peanuts.

The sensory characteristics of four peanut varieties, both raw and boiled, were evaluated according to established sensory evaluation criteria, as shown in Fig. 1. The overall acceptability of fresh peanuts (ranging from 8.32 to 8.68) was found to be higher than that of boiled peanuts (ranging from 6.33 to 7.83). Notably, Sample II (Jihuatian 1) exhibited the highest overall acceptability both before and after boiling, with scores of 8.68 for fresh peanuts and 7.83 for boiled peanuts, indicating that this variety is particularly suitable for boiling. From a holistic perspective, Sample II demonstrated superior acceptability in terms of both raw and boiled forms, suggesting its potential as the preferred choice for boiled peanut production. In terms of physical and visual attributes, no significant differences were observed in the color, size, or shape of the peanut pods before and after boiling. However, the boiled peanut kernels exhibited marked improvements in color, flavor, and brittleness compared to their raw counterparts. These changes can be attributed to the chemical interactions between proteins, lipids, and polysaccharides during boiling, which likely stimulate the release of flavor compounds. Additionally, the boiling process leads to alterations in the cellular structure of the peanuts, contributing to shifts in both flavor and texture. Regarding flavor profile, the boiled peanuts, particularly those from Sample II (fresh peanuts), maintained a distinct advantage (score: 7.67) over their raw counterparts (Sobolev, 2001). However, it was observed that the texture of raw peanuts was rated higher than that of the boiled peanuts. The boiling process may increase the relative fiber content within the peanuts, which in turn may negatively affect the perception of texture, leading to a decrease in the fine texture and, consequently, a reduction in the overall acceptability of the boiled peanuts' texture. Therefore, the boiling process enhances the flavor, color, brittleness, and overall sensory qualities of the peanut pods and kernels, it simultaneously introduces a reduction in texture quality due to changes in the cellular structure and an increase in fiber content.

Note:Data are presented as mean ± standard deviation (*n* = 3). II (Jihuatian 1) exhibited the highest overall acceptability in both raw (8.68) and boiled (7.83) states, indicating its superior suitability for boiling; boiled peanuts showed increased flavor/brittleness scores but decreased texture fineness scores compared to raw counterparts.

### 3.2 Nutritional content

**Fig. 2:**
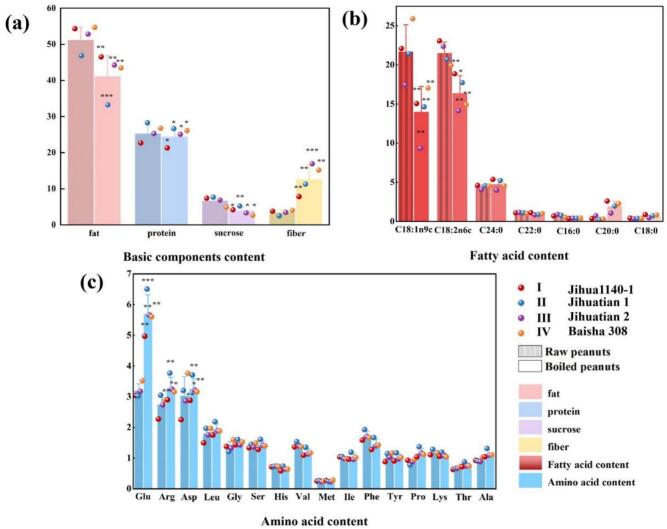
Effect of boiling on the nutritional quality of different peanut varieties.

As can be seen from Fig. 2, the variation range of fat content of the 4 raw materials was 42.19–50.69 g/100 g, and the minimum content was sample IIY. Among the fatty acids measured, the contents of oleic acid and linoleic acid were the highest, and the contents of oleic acid ranged from 15.97 to 24.44 g/100 g, and the contents of linoleic acid ranged from 18.82 to 21.39 g/100 g, and the contents of linoleic acid were the highest in sample IY. The sucrose content varied from 4.56 to 6.95 g/100 g, which was similar to the results of Wang's study (2018), and the maximum content was in sample IIY. The range of crude fiber content was 2.30–3.80 g/100 g, and the minimum content was sample IIY. Protein content ranged from 20.69 to 25.49 g/100 g, which was consistent with the results of Gong (2018) and Nayak et al. (2020), and the maximum protein content was in sample IIY. The contents of arginine (Arg), aspartate (Asp) and glutamic acid (Glu) were the highest among the amino acids measured. The variation range of arginine was 2.11–2.80 g/100 g, that of aspartate was 2.09–3.56 g/100 g, and that of glutamic acid was 2.78–3.33 g/100 g. The fat content of the 4 boiled peanuts ranged from 16.41 to 26.29, and the lowest content was in sample IIS. The content of oleic acid varied from 4.68 to 10.48 g/100 g, and the content of linoleic acid varied from 7.09 to 10.02 g/100 g. The content of saturated fatty acids was the lowest in sample IIS, and the content of sucrose was the highest in sample IIS. The minimum crude fiber content was in sample IS. The maximum protein content was IVS.

After boiling, the fat content decreased by 19.73%, the protein content decreased by 3.76%, and the decrease in protein content after boiling may be related to the denaturation and solublization of certain nitrogen-containing compounds (such as amides and amines) during processing. Sucrose content was significantly reduced by 42.21%, and the main reason for the reduction in sucrose content was leaching into water and decomposition into glucose and fructose (Aljahdali & Carbonero, 2019; de Camargo et al., 2017; Xu et al., 2014). The crude fiber content was significantly increased by 72.73%, which may result in a lower score of taste fineness. The content of unsaturated fatty acids decreased significantly, with oleic acid content dropping by 35.50% and linoleic acid content by 23.83%. This is due to the oxidation and decomposition of unsaturated fatty acids caused by boiling, which is similar to the research conclusion of Ma, Zhang, Xu, and Zhao (2024). The contents of arachidic acid and stearic acid in saturated fatty acids increased by 52.24% and 83.25%. The content of most amino acids decreased significantly, the decrease of glutamic acid and arginine was 43.93% and 16.21% respectively. When the boiling temperature was 100 °C, the amount of amino acid Maillard reaction was low, and most amino acids had high water solubility, so the loss of content may be caused by the dissolution of amino acids.

Fig. 2 shows the basic component contents, fatty acid contents and amino acid contents of different varieties of peanuts before and after boiling. *p < 0.05, **p < 0.01, ***p < 0.001. No significant differences are marked (marked as differences between raw and boiled groups of the same variety).

### 3.3 Texture characteristics

The hardness range of the four raw material peanut samples was 6335.40–7942.97 g, in which the hardness of sample IY was the highest, the hardness of sample IVY was the lowest, and the brittleness range was 1.26–1.42 mm, among which the brittleness of sample IIY was the highest and the brittleness of sample IY was the least. The hardness of the 4 boiled peanut samples ranged from 4594.28 to 6061.16 g, in which the hardness of sample IIIS was the highest, the hardness of sample IVS was the lowest, and the brittleness value ranged from 2.10 to 2.46 mm, among which the brittleness of sample IIIS was the highest and the brittleness of sample IIS was the least.

Texture characteristics will affect the taste of peanuts. After boiling, the hardness value decreased by 49.11%, while the brittleness value increased by 59.85%. This is consistent with the findings of Smith and Raghavan (2025), who discovered that baking softens peanuts by breaking the non-covalent bonds in the proteins. During the boiling process, water enters the peanut, the water content of the peanut increased, the structure was destroyed, and the brittleness of the peanut decreased. The paste structure appeared inside the peanut, the protein denaturation, starch gelatinization and the damage of surface cells caused the hardness of the peanut to decrease.

**Fig. 3:**
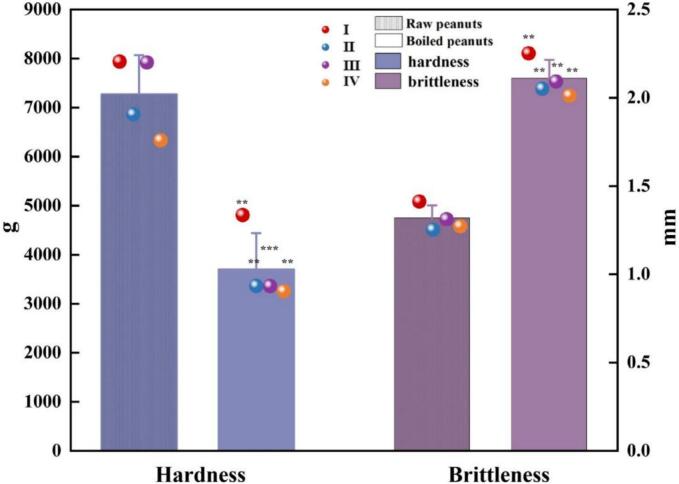
Analysis of texture characteristics of peanuts from different raw materials and boiled peanuts.

Fig. 3 shows the hardness and brittleness of different raw peanuts and boiled peanuts. *p < 0.05, **p < 0.01, ***p < 0.001. No significant differences are marked (marked as differences between raw and boiled groups of the same variety).

### 3.4 Volatile flavor components of different varieties of fresh peanuts

**Fig. 4:**
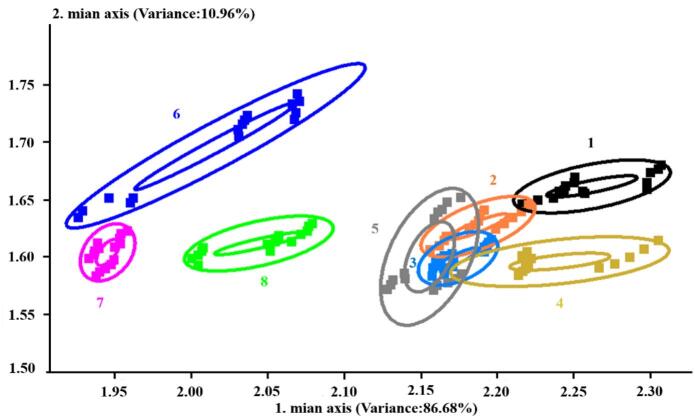
PCA analysis of different varieties of raw and boiled peanuts.

Fig. 4 showed the distribution of 15 parallel detection data of different peanut samples on the PCA plane. The contribution rate of the first principal component was 86.68%, the contribution rate of the second principal component was 10.96%, and the cumulative contribution rate was 97.64%. In the samples of 4 different raw materials, there were significant differences in flavor pairs. There were significant differences in flavor pairs among 4 different boiled peanut samples, among which there were significant differences between IS and IIS samples, and there were significant differences between IS and IIIS samples. The distance between raw peanut samples of IY ∼ IVY and boiled peanut samples of IIS ∼ IVS was large. In order to further clarify the changes of flavor components, the volatile flavor substances were further analyzed and identified by GC–MS (Table Supplement 1).

The 58 volatile components were detected from 4 different peanut raw materials. Including 19 esters accounting for 30.05%, 9 alcohols 6.60%, 8 alkanes 14.79%, 5 ketones 23.00%, 4 aldehydes 1.46%, 3 acids 7.61%, 2 phenols 7.66%, 1 furan 1.97%, 1 pyrrole 0.42%, 1 olefin 0.64%, 1 thiazole 2.68%, 4 other 3.14%. Different varieties of peanut raw material flavor substance types were basically the same, but the composition content was different. The total amount of esters ranged from 143.47 to 341.84 μg/kg, alcohols from 31.08 to 72.24 μg/kg, alkanes from 59.43 to 168.58 μg/kg, ketones from 114.77 to 250.16 μg/kg. Aldehydes were in the range of 6.00–17.97 μg/kg, acids were in the range of 16.02–177.17 μg/kg, phenols were in the range of 23.06–106.94 μg/kg, furans were in the range of 8.34–22.48 μg/kg, pyrroles were in the range of 0.86–4.82 μg/kg. Olefin 2.82–6.42 μg/kg, thiazole 7.18–41.36 μg/kg, other compounds total 9.30–72.33 μg/kg. There were 11 kinds of characteristic flavor substances, as shown in Table 2, including 2 alcohols, 3 aldehydes, 2 esters, 1 ketone, 1 furan, 1 olefin and 1 other. The types of characteristic flavor substances of peanut raw materials of different varieties were significantly different, and the volatile components of IIY and IIIY samples were the highest.

**Table 2:** OAV values of characteristic flavor substances of four different peanut raw materials.

Compounds	CAS	Odor description	I	II	III	IV
2,4-Decadienal,(E,E)-	25152-84-5	Cucumbernut	22.56	87.86	85.49	32.21
Decanoicacid,methylester	110-42-9	Fruityfloral	11.55	13.62	14.15	31.27
Hexanal	66-25-1	Freshgreen	8.98	15.53	21.07	16.60
Benzeneacetaldehyde	122-78-1	Greenfloral	6.47	10.23	24.44	18.46
1-Hexanol	111-27-3	Fruitygreen	1.79	2.09	6.14	4.69
PhenylethylAlcohol	60-12-8	Flower	2.82	3.24	3.75	3.55
Furan,2-pentyl-	3777-69-3	Greenearthybeany	1.74	4.68	3.56	3.39
2-Octanone	111-13-7	Earthyherbal	1.64	3.00	3.56	1.62
Limonene	138-86-3	Herbal	<1	1.60	1.45	1.41
Naphthalene	91-20-3	Pungentdrytarry	<1	1.24	1.02	1.00
Butanoicacid,2-methyl-,ethylester	7452-79-1	Greenfruity	<1	<1	2.29	<1

A total of 54 kinds of flavor substances were detected in 4 different varieties of boiled peanuts. It included 16 esters 28.61%, 9 alcohols 8.25%, 8 alkanes 15.47%, 5 ketones 3.49%, 4 aldehydes 19.85%, 2 acids 1.90%, 2 phenols 3.18%, 1 furan 4.29%, 1 pyrrole 11.44%, 1 olefin 0.81%, and 1 mesthiazole 0.42% and 5 other 2.29% (See Table 3). Compared with raw peanuts, boiled peanuts lack three esters and one acid flavor substance. Because the heating temperature of the boiling water was relatively mild, the volatile flavor substances produced were relatively small, and the types were not different from the raw peanuts. The total amount of esters ranged from 181.52 to 307.14 μg/kg, alcohols from 63.16 to 86.42 μg/kg, alkanes from 77.12 to 229.53 μg/kg, ketones from 18.22 to 44.44 μg/kg. The contents of aldehydes ranged from 124.99 to 315.52 μg/kg, acids from 5.40 to 26.59 μg/kg, phenols from 5.37 to 44.36 μg/kg, furans from 28.55 to 52.70 μg/kg, pyrroles from 66.09 to 130.10 μg/kg. Olefin 4.11–12.05 μg/kg, thiazole 2.93–5.6 μg/kg, other compounds total 18.98–30.75 μg/kg. There were 10 kinds of characteristic flavor substances, as shown in Table 2, including 2 alcohols, 3 aldehydes, 2 esters, 1 furan, 1 thiazole, and 1 other. There were significant differences in the content of characteristic flavor substances among different varieties of boiled peanut. The volatile components of IIIS and IVS samples were higher and the flavor was more intense.

**Table 3:** OAVvaluesofcharacteristicflavorsubstancesoffourdifferentboiledpeanuts.

Compounds	CAS	Odordescription	I	II	III	IV
2,4-Decadienal,(E,E)-	25152-84-5	Cucumbernut	377.65	391.52	340.22	280.73
Benzeneacetaldehyde	122-78-1	Greenfloral	73.23	116.67	324.21	770.48
Hexanal	66-25-1	Freshgreen	289.71	265.22	169.31	239.63
Butanoicacid,2-methyl-,ethylester	7452-79-1	Greenfruity	1.13	17.77	18.59	<1
Furan,2-pentyl-	3777-69-3	Greenearthybeany	5.95	10.98	8.19	6.82
PhenylethylAlcohol	60-12-8	Flower	5.11	8.19	6.18	6.53
1-Hexanol	111-27-3	Fruitygreen	2.89	4.82	1.46	4.10
Decanoicacid,methylester	110-42-9	Fruityfloral	1.65	2.28	4.10	2.79
Limonene	138-86-3	Herbal	1.27	1.87	3.01	1.03

Compared with peanut raw material, boiled peanut had one less characteristic flavor substance, which was para-octanone, and the flavor was expressed as earthy and herbaceous. Compared with boiled peanuts, roasted peanuts have a greater variety of volatile aromas and a more intense aroma (Zhang, Zhu, Jin, Xu, & Chen, 2024). Aldehydes were important non-heterocyclic volatile components in the flavor substances of boiled peanuts. Fat degradation produced hexanal (Zhang, Shen, Cao, Duan, & Sun, 2023), the flavor expression of hexanal was fresh and green, and the degradation of aromatic amino acid phenylalanine in peanuts produced phenylacetaldehyde. The aroma of benzeneacetaldehyde was expressed as green and floral. 2, 4-decadienal,(E,E)- was expressed as cucumber and nut. Aldehydes gave boiled peanuts a special nutty aroma and a sweet, powdery texture. The aldehydes in boiled peanuts were derived from the oxidative decomposition of unsaturated fatty acids. Specifically, the formation of aldehydes during peanut boiling mainly follows two key pathways of fatty acid oxidation: enzymatic oxidation and non-enzymatic autoxidation (Chukwumah et al., 2007; Zhang et al., 2023). For enzymatic oxidation, the lipoxygenase (LOX) naturally present in fresh peanuts remains active in the initial stage of boiling, and it specifically catalyzes the oxidation of polyunsaturated fatty acids (e.g., linoleic acid, C18:2, and α-Linolenic acid, C18:3) with cis,cis-1,4-pentadiene structures. This catalysis generates hydroperoxides as intermediates. As the boiling process continues, the thermal inactivation of LOX (above 80 °C) inhibits enzymatic reactions, while non-enzymatic autoxidation becomes the dominant pathway. The hydroperoxides generated in the early stage undergo thermal decomposition under boiling temperature (100 °C), and break down into short-chain aldehydes (e.g., hexanal) via homolytic cleavage of the O—O bond in hydroperoxides, or into unsaturated aldehydes (e.g., (E,E)-2,4-decadienal) via cleavage of the C—C bond adjacent to the hydroperoxide group (Zhang et al., 2023). Additionally, the aromatic aldehyde detected in boiled peanuts is not only derived from the oxidation of unsaturated fatty acids but also from the thermal degradation of aromatic amino acids (e.g., phenylalanine) (Hu et al., 2021), which further enriches the aldehyde profile of boiled peanuts. This multipathway oxidation of fatty acids and amino acids jointly explains the significant increase in aldehyde content observed in this study, and also leads to changes in its microstructure.

The peanut sample also contained pleasant limonene, which provided the sweetness of fresh fruit. The content of raw peanuts was 2.82–6.42 μg/kg, the content of limonene in sample IIY was the highest, the content of boiled peanuts was 4.11–12.05 μg/kg, and the content of limonene in sample IIIS was the highest. The content of sucrose and free amino acids was crucial to the formation of flavor substances (Baker et al., 2003; Simkovic, Surina, & Vrican, 2003). During the boiling process, sugar degradation and Maillard reaction occured in the sucrose of peanuts, and the 2-amylfuran produced provided sweetness, nutty aroma and bean aroma (Berk, Aktag, & Gokmen, 2021; Hu et al., 2021). The content of raw peanut species was 8.34–22.48 μg/kg, and the content of 2-amylfuran in sample IIY was the highest. The contents of boiled peanuts ranged from 28.55 to 52.70 μg/kg, and the contents of 2-amyl furan in sample IIS were the highest, which may be caused by the degradation of sugar. So boiled peanuts tasted fragrant, soft waxy, powder sweet.

Note: Clear separation was observed between raw and boiled samples (large distance between IY ∼ IVY and IIS ∼ IVS), indicating significant flavor changes after boiling; among boiled samples, IS (Jihua 1140–1) showed distinct flavor profiles from IIS (Jihuatian 1) and IIIS (Jihuatian 2).

### 3.5 Microstructure of fresh peanuts

**Fig. 5:**
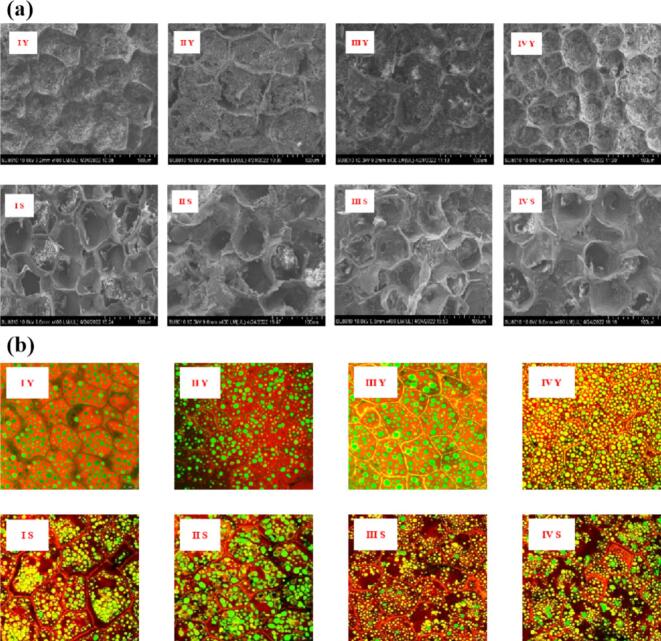
SEM (400×) and CLSM (40×) were used to observe the microstructure of different raw peanuts and boiled peanuts.

From the microstructure, the cell size, shape, protein body size, distribution and cell wall thickness of different varieties of peanut were different. Scanning electron microscopy (SEM) (Fig. 5a) showed that the raw peanut cells were complete in structure, and the outer layer was composed of a single layer of epidermal cells and parenchyma cells. The particles in the parenchyma cells were oil bodies, protein bodies and starch particles, and the particles were full and round. The edible peanuts of sample II and sample III have large single-cell structure, in which the protein or starch globular particles can be clearly seen in the cross-section of peanut of sample II. The cell walls of peanuts I and IV are thick, and the single cell section can clearly see the bright spots of oil in a dispersed state (Li et al., 2025b; Xie et al., 2023). The diameter of the protein body was 5–12 μm, the diameter of the starch particle was 4–15 μm, and the diameter of the oil body was about 1–2 μm. The membrane of the particles formed a continuous, dense cytoplasmic network.

Notably, the intrinsic microstructural traits of Jihuatian 1 (sample IIY) further underpin its superior boiling suitability, with large single-cell structures, uniformly distributed protein bodies and starch granules, and moderately thick cell walls facilitating efficient water penetration during boiling. This induces sufficient cell wall rupture and moderate cytoplasm loss, avoiding excessive nutrient leaching or inadequate structural softening. Complementarily, Jihuatian 1 exhibits the highest initial sucrose and protein contents, the lowest crude fiber content (Section 3.2), its high sucrose content serving as abundant precursors for the Maillard reaction and flavor compound formation (e.g., 2-pentylfuran) while moderate protein content and uniform protein body distribution underpin optimal textural softening during processing.

After boiling, the microstructure of peanut changes. The changes of peanut structure were mainly reflected in the appearance of paste structure in the inside, damage of surface cells and loss of cytoplasm. As shown in Fig. 5, it can be seen that the cytoplasm in some cells was almost completely lost, which was due to the serious destruction of the cell layer on the surface of the peanut after boiling, resulting in the dissolution of the cytoplasmic components into the soup. In addition, it can be observed that there was a mushy structure inside the peanut, which was caused by the large amount of water entering the peanut and the gelatinization of protein-modified starch due to high boiling temperature (Young, Pattee, Schadel, & Sanders, 2004).

By confocal laser microscopy (CLSM, Fig. 5b), it can be observed that the cell structure is partially destroyed, the cell expands, the protein body distributes and aggregates, and the protein body, oil and other nutrients are lost. The cell wall was broken to different degrees, causing the original regular distribution of cell structure to be partially destroyed, showing a more chaotic distribution. Under the same observation multiple, the number of cells decreased, the volume of cells increased, the uniform distribution of proteomes gathered, and the thickness of the cell wall decreased. The results observed by CLSM and SEM were consistent. The SEM image showed that the surface cells of peanut were damaged, the inside of peanut appeared paste structure, and the cytoplasm was lost. The CLSM image showed that the cell wall was broken, water entered the cell and expanded, the cytoplasm was lost, and the protein content inside peanut was reduced and aggregated. The protein aggregation observed in CLSM images (Fig. 5b) is essentially a consequence of thermal denaturation of peanut proteins, which further drives the textural changes of boiled peanuts (Li et al., 2024; Wang et al., 2022). During boiling, the thermal energy disrupts the non-covalent bonds (hydrogen bonds, hydrophobic interactions, and electrostatic interactions) that maintain the native tertiary and quaternary structures of peanut proteins. This denaturation exposes the hydrophobic groups originally buried in the protein interior, leading to intermolecular aggregation of denatured proteins via hydrophobic interactions and disulfide bond cross-linking (Yang et al., 2025). Additionally, the thermal denaturation of proteins also promotes the gelatinization of starch (by reducing the interaction between proteins and starch granules), and the synergistic effect of protein aggregation and starch gelatinization further enhances the soft and waxy mouthfeel of boiled peanuts, which is consistent with the sensory evaluation results (3.1) that boiled peanuts have improved brittleness but reduced texture fineness.

### 3.6 Correlation analysis between raw materials of fresh peanuts and quality of boiled peanuts

**Fig. 6:**
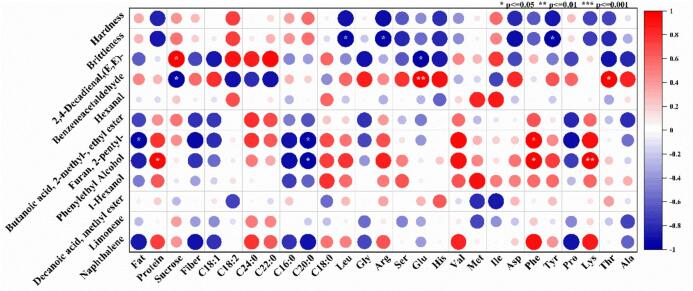
Correlation between different raw materials of fresh peanuts and the quality of boiled peanuts.

In order to explore the correlation between raw materials of fresh peanuts and the quality of boiled peanuts, correlation analysis was carried out between the quality indexes of 27 peanut raw materials and the texture and characteristic flavor substances of boiled peanuts, and the results were shown in Fig. 6. The structural properties (hardness and brittleness) of boiled peanut were negatively correlated with protein content and amino acid content, but positively correlated with sucrose and fatty acid content. This may be because the lubrication of oil had a positive effect on the texture of boiled peanuts. Interestingly, the correlations of 2, 4-decadienal (E,E)- and benzeneacetaldehyde with the quality of boiled peanuts were reversed. Among them, 2, 4-decadienal (E,E)- was significantly positively correlated with sucrose, and with sucrose of C18:2, C24:0 and C22:0. In contrast, benzeneacetaldehyde was positively correlated with C18:1, Glu, Thr, Gly, His and Asp, and negatively correlated with many fatty acids. This may be because the formation of these two flavor substances comes from the oxidative decomposition of fatty acids, and the other comes from the Maillard reaction of amino acids. More in-depth mechanisms need to be analyzed by more systematic omics techniques. Furan, 2-pentyl- showed significant positive correlation with Phe and Lys, Val, Protein, etc., indicating that the Maillard reaction of amino acids may contribute a lot to this flavor. On the contrary, Furan, 2-pentyl- showed a significant negative correlation with Fat and C20:0, and a significant negative correlation with Fiber, C18:1, C16:0 and Pro. The correlation between Phenylethyl Alcohol and Furan, 2-pentyl- is generally consistent. There was a close correlation between the flavor of boiled peanuts and the quality of raw materials, most of which were related to the content of fatty acids, sucrose and amino acids. This also showed that the raw material quality of different varieties of fresh peanuts had a significant effect on the texture and flavor of their boiled peanuts.

## 4 Conclusion

Different varieties of fresh peanuts undergo significant quality changes during boiling, with Jihuatian 1 standing out as the optimal raw material due to its highest sucrose and crude fiber content both before and after processing, along with superior texture and sensory acceptability after boiling. Boiling destroys the cell structure of peanuts, causing damage to the cell surface, partial rupture of the cell wall and loss of cytoplasm, promoting the leaching of nutrients and creating conditions for biochemical reactions. Lipid oxidation, initially catalyzed by active lipoxygenase and later dominated by non-enzymatic autoxidation, reduces unsaturated fatty acids while generating aldehyde-type flavor compounds, which increase from 1.46% to 19.85% of total volatiles and contribute to the characteristic sweet and nutty aroma. Meanwhile, protein denaturation and starch gelatinization synergistically reduce hardness by 49.11% and increase brittleness by 59.85%, altering mouthfeel, and the Maillard reaction between reduced sugars and amino acids enriches flavor via compounds like 2-pentylfuran. Correlation analysis confirms that raw material traits—including fatty acid composition, sucrose, and amino acid levels—closely influence boiled peanut texture and flavor. These findings clarify the intrinsic links between boiling-induced microstructural, nutritional, and flavor changes, providing theoretical and practical guidance for the peanut processing industry to optimize variety selection and processing parameters, supporting the development of high-quality boiled peanut products that meet consumer demands for taste, texture, and nutrition.

## CRediT authorship contribution statement

**Xuegang Huang:** Writing – original draft, Visualization, Validation, Software, Formal analysis. **Zhenyuan Li:** Writing – original draft, Visualization, Validation, Data curation. **Gengjiu Zhao:** Writing – review & editing, Supervision, Conceptualization. **Yumeng Hu:** Writing – review & editing. **Fengying Gu:** Writing – review & editing, Supervision, Project administration. **Qiang Wang:** Writing – review & editing, Supervision, Funding acquisition, Conceptualization. **Qin Guo:** Writing – review & editing, Resources, Conceptualization.

## Ethical statement

Ethical permission, to conduct a human sensory study, was granted by Institute of Food Science and Technology, Chinese Academy of Agricultural Sciences. The ethical and professional requirements of the IFST (*the Institute of Food Science & Technology, UK*) were strictly adhered to in the sensory evaluation of the study, all participants voluntarily agreed to participate in the sensory study and signed a written consent statement. They fully understood the requirements and risks of the study, their rights and privacy were protected, and they could withdraw at any time.

## Declaration of competing interest

The authors declare that they have no known competing financial interests or personal relationships that could have appeared to influence the work reported in this paper.

## Data Availability

Data will be made available on request.
