# Supplementary material for: Effect of boiling on quality, microstructure and flavor of fresh peanuts
Source: Food Chem X. 2026 Feb 3;34:103632. doi: 10.1016/j.fochx.2026.103632 (PMC12915249; doi:10.1016/j.fochx.2026.103632)
Supplement: Supplementary material [file mmc1.docx]

**Supplement 1**

**4 raw materials peanut volatile flavor components content**

| compounds | CAS | Ⅰ | Ⅱ | Ⅲ | Ⅳ |
| --- | --- | --- | --- | --- | --- |
| 1-Butanol, 3-methyl- | 123-51-3 | 0.31 | 0.54 | 0.99 | 0.17 |
| 1-Hexanol | 111-27-3 | 10.03 | 11.69 | 34.39 | 26.28 |
| 1-Hexanol, 2-ethyl- | 104-76-7 | 1.72 | 4.37 | 3.61 | 3.72 |
| 1-Octanol | 111-87-5 | 1.44 | 4.68 | 5.93 | 7.10 |
| 1-Pentanol | 71-41-0 | 1.75 | 1.94 | 4.52 | 4.09 |
| 3-Decen-1-ol,(Z)- | 10340-22-4 | 0.88 | 1.89 | 0.77 | 0.70 |
| 2-Nonen-1-ol | 22104-79-6 | 4.09 | 8.05 | 3.95 | 5.82 |
| Benzyl alcohol | 100-51-6 | 1.92 | 5.02 | 6.20 | 3.50 |
| Phenylethyl Alcohol | 60-12-8 | 8.95 | 10.29 | 11.89 | 11.26 |
| Butanoic acid, 2-methyl-, ethyl ester | 7452-79-1 | - | 0.06 | 0.34 | - |
| Decanoic acid, methyl ester | 110-42-9 | 1.85 | 2.18 | 2.26 | 5.00 |
| 2(3H)-Furanone, dihydro-5-pentyl- | 104-61-0 | 1.69 | 4.11 | 3.26 | 2.43 |
| Dibutyl phthalate | 84-74-2 | 19.55 | 31.58 | 37.66 | 59.66 |
| 2H-Pyran-2-one, tetrahydro- | 542-28-9 | 3.65 | 2.23 | 2.18 | 2.01 |
| Heptanoic acid, methyl ester | 106-73-0 | 2.98 | 2.60 | 2.77 | 1.90 |
| Hexadecanoic acid, methyl ester | 112-39-0 | 5.08 | 13.03 | 15.06 | 7.32 |
| Dimethyl phthalate | 131-11-3 | 0.97 | 2.33 | 2.16 | 1.22 |
| Nonanoic acid, methyl ester | 1731-84-6 | 6.96 | 9.67 | 5.86 | 3.82 |
| Octanoic acid, methyl ester | 111-11-5 | 8.62 | 7.32 | 7.22 | 19.52 |
| Methyl tetradecanoate | 124-10-7 | 0.83 | 1.67 | 2.04 | 0.76 |
| Methyl valerate | 624-24-8 | 1.49 | 1.35 | 1.53 | 1.25 |
| Hexanoic acid, methyl ester | 106-70-7 | 27.91 | 26.81 | 34.24 | 27.67 |
| 3-Methylcyclopentyl acetate | 24070-70-0 | 1.52 | 0.64 | 0.62 | 0.53 |
| Carbonic acid, nonyl vinyl ester | - | 1.17 | 3.91 | 4.88 | 7.04 |
| 1,2-Benzenedicarboxylic acid, bis(2-methylpropyl) ester | 84-69-5 | 54.29 | 93.15 | 112.81 | 170.32 |
| Oxalic acid, allyl dodecyl ester | 959312-46-0 | 3.47 | 13.58 | 15.93 | 18.06 |
| Oxalic acid, isohexyl neopentyl ester | - | 1.25 | 4.08 | 5.43 | 12.83 |
| trans-13-Octadecenoic acid, methyl ester | 42199-38-2 | 0.18 | 0.51 | 1.09 | 0.50 |
| 2,6-Di-tert-butyl-4-hydroxy-4-methylcyclohexa-2,5-dien-1-one | 10396-80-2 | 9.14 | 17.09 | 11.64 | 3.06 |
| 4-Butanol-2-one | 590-90-9 | - | 0.56 | 5.47 | 0.76 |
| 2-Octanone | 111-13-7 | 65.69 | 119.92 | 142.55 | 65.00 |
| 2-Propanone, 1-hydroxy- | 116-09-6 | 39.21 | 89.98 | 86.82 | 67.71 |
| 2-Nonen-4-one | 32064-72-5 | 0.72 | 3.30 | 3.69 | 10.51 |
| 3,5-di-tert-Butyl-4-hydroxybenzaldehyde | 1620-98-0 | 0.58 | 1.74 | 1.58 | 0.56 |
| Hexanal | 66-25-1 | 2.87 | 4.97 | 6.74 | 5.31 |
| Benzeneacetaldehyde | 122-78-1 | 1.94 | 3.07 | 7.33 | 5.54 |
| 2,4-Decadienal,(E,E)- | 25152-84-5 | 0.61 | 2.37 | 2.31 | 0.87 |
| Cycloheptasiloxane, tetradecamethyl- | 107-50-6 | 53.64 | 125.73 | 85.39 | 121.09 |
| Decane, 2,3,7-trimethyl- | 62238-13-5 | 0.58 | 1.99 | 1.57 | 1.57 |
| Pentadecane | 629-62-9 | 1.32 | 3.20 | 3.18 | 4.57 |
| Tetradecane | 629-59-4 | 0.32 | 1.17 | 1.39 | 3.12 |
| Undecane, 2-methyl- | 7045-71-8 | 0.66 | 3.40 | 4.39 | 11.93 |
| Undecane, 4-methyl- | 2980-69-0 | 1.13 | 5.70 | 7.09 | 18.69 |
| Decane, 5-propyl- | 17312-62-8 | 1.14 | 2.66 | 3.07 | 3.23 |
| Dodecane, 4,6-dimethyl- | 61141-72-8 | 0.64 | 1.38 | 2.28 | 4.39 |
| 2,4-Di-tert-butylphenol | 96-76-4 | 0.98 | 2.39 | 1.92 | 0.93 |
| Butylated Hydroxytoluene | 128-37-0 | 22.08 | 103.44 | 105.02 | 27.65 |
| Hexanoic acid | 142-62-1 | 0.53 | 2.07 | 4.20 | 2.15 |
| Propanoic acid, 2-oxo- | - | 10.20 | 30.98 | 29.65 | 32.49 |
| L-Lactic acid | 79-33-4 | 5.28 | 9.63 | 143.32 | 7.52 |
| Naphthalene, 1,6,7-trimethyl- | 2245-38-7 | 1.69 | 6.32 | 5.91 | 2.37 |
| Naphthalene, 1-methyl- | 90-12-0 | 0.64 | 2.54 | 2.26 | 2.40 |
| Naphthalene | 91-20-3 | 2.32 | 11.17 | 9.16 | 9.04 |
| Benzene, 1,4-dichloro- | 106-46-7 | 4.64 | 18.04 | 13.65 | 14.31 |
| Benzothiazole | 95-16-9 | 7.18 | 28.42 | 41.36 | 16.56 |
| Furan, 2-pentyl- | 3777-69-3 | 8.34 | 22.48 | 17.07 | 16.30 |
| Limonene | 138-86-3 | 2.82 | 6.42 | 5.81 | 5.65 |
| 1H-Pyrrole, 1-methyl- | 96-54-8 | 4.82 | 0.86 | 1.57 | 3.45 |

**Volatile flavor component content of 4 kinds of boiled peanuts**

| compounds | CAS | Ⅰ | Ⅱ | Ⅲ | Ⅳ |
| --- | --- | --- | --- | --- | --- |
| 1-Butanol, 3-methyl- | 123-51-3 | 1.08 | 3.24 | 2.97 | 0.40 |
| 1-Hexanol | 111-27-3 | 16.21 | 27.00 | 8.20 | 22.97 |
| 1-Hexanol, 2-ethyl- | 104-76-7 | 3.52 | 2.36 | 2.26 | 2.41 |
| 1-Octanol | 111-87-5 | 1.51 | 1.86 | 1.43 | 1.38 |
| 1-Pentanol | 71-41-0 | 3.35 | 3.33 | 2.16 | 4.11 |
| 2-Nonen-1-ol | 22104-79-6 | 30.66 | 19.01 | 21.28 | 13.41 |
| Benzyl alcohol | 100-51-6 | 2.47 | 2.34 | 3.86 | 2.84 |
| Phenylethyl Alcohol | 60-12-8 | 16.20 | 25.97 | 19.59 | 20.70 |
| 3-Decen-1-ol,(Z)- | 10340-22-4 | 1.48 | 1.32 | 1.41 | 0.81 |
| Butanoic acid, 2-methyl-, ethyl ester | 7452-79-1 | 0.17 | 2.67 | 2.79 | - |
| Decanoic acid, methyl ester | 110-42-9 | 0.26 | 0.36 | 0.66 | 0.45 |
| 2(3H)-Furanone, dihydro-5-pentyl- | 104-61-0 | 0.84 | 1.92 | 1.13 | 1.11 |
| Dibutyl phthalate | 84-74-2 | 74.33 | 67.95 | 60.87 | 44.58 |
| 2H-Pyran-2-one, tetrahydro- | 542-28-9 | 1.81 | 1.50 | 1.80 | 0.80 |
| Hexadecanoic acid, methyl ester | 112-39-0 | 0.47 | 0.53 | 0.43 | 0.53 |
| Dimethyl phthalate | 131-11-3 | 0.41 | 0.32 | 0.60 | 0.25 |
| Nonanoic acid, methyl ester | 1731-84-6 | 0.06 | 0.10 | 0.19 | 0.13 |
| Octanoic acid, methyl ester | 111-11-5 | 0.55 | 0.99 | 2.32 | 1.72 |
| Methyl tetradecanoate | 124-10-7 | 0.10 | 0.08 | 0.11 | 0.18 |
| Hexanoic acid, methyl ester | 106-70-7 | 0.11 | 0.19 | 0.16 | 0.15 |
| 3-Methylcyclopentyl acetate | 24070-70-0 | 0.76 | 0.55 | 0.62 | 0.44 |
| Carbonic acid, nonyl vinyl ester | - | 2.52 | 2.87 | 2.77 | 2.43 |
| 1,2-Benzenedicarboxylic acid, bis(2-methylpropyl) ester | 84-69-5 | 219.97 | 193.83 | 167.42 | 124.20 |
| Oxalic acid, allyl dodecyl ester | 959312-46-0 | 2.10 | 3.38 | 3.30 | 2.14 |
| Oxalic acid, isohexyl neopentyl ester | - | 2.66 | 2.44 | 2.58 | 2.43 |
| 2,6-Di-tert-butyl-4-hydroxy-4-methylcyclohexa-2,5-dien-1-one | 10396-80-2 | 3.89 | 2.45 | 3.60 | 0.86 |
| 4-Butanol-2-one | 590-90-9 | 5.11 | 29.40 | 9.42 | - |
| 2-Octanone | 111-13-7 | 3.84 | 5.66 | 4.58 | 6.80 |
| 2-Propanone, 1-hydroxy- | 116-09-6 | 4.71 | 5.73 | 8.86 | 22.74 |
| 2-Nonen-4-one | 32064-72-5 | 0.67 | 1.19 | 1.24 | 1.90 |
| 3,5-di-tert-Butyl-4-hydroxybenzaldehyde | 1620-98-0 | 0.12 | 0.13 | 0.20 | 0.12 |
| Hexanal | 66-25-1 | 92.71 | 84.87 | 54.18 | 76.68 |
| Benzeneacetaldehyde | 122-78-1 | 21.97 | 35.00 | 97.26 | 231.14 |
| 2,4-Decadienal,(E,E)- | 25152-84-5 | 10.20 | 10.57 | 9.19 | 7.58 |
| Cycloheptasiloxane, tetradecamethyl- | 107-50-6 | 131.24 | 60.55 | 106.89 | 218.17 |
| Decane, 2,3,7-trimethyl- | 62238-13-5 | 0.61 | 1.04 | 1.09 | 0.74 |
| Pentadecane | 629-62-9 | 2.83 | 5.03 | 3.84 | 2.68 |
| Tetradecane | 629-59-4 | 1.03 | 1.46 | 1.16 | 1.40 |
| Undecane, 2-methyl- | 7045-71-8 | 1.10 | 0.88 | 0.98 | 1.13 |
| Undecane, 4-methyl- | 2980-69-0 | 1.16 | 1.45 | 1.51 | 1.77 |
| Decane, 5-propyl- | 17312-62-8 | 2.18 | 3.55 | 3.33 | 2.55 |
| Dodecane, 4,6-dimethyl- | 61141-72-8 | 1.07 | 3.16 | 1.40 | 1.08 |
| 2,4-Di-tert-butylphenol | 96-76-4 | 1.19 | 1.16 | 1.60 | 0.67 |
| Butylated Hydroxytoluene | 128-37-0 | 17.21 | 42.39 | 42.77 | 4.70 |
| Propanoic acid, 2-oxo- | - | 2.82 | 8.33 | 11.00 | - |
| L-Lactic acid | 79-33-4 | 10.40 | 18.27 | 8.92 | 5.40 |
| Benzene, 1,4-dichloro- | 106-46-7 | 8.68 | 7.82 | 11.66 | 7.92 |
| Naphthalene, 1,6,7-trimethyl- | 2245-38-7 | 1.80 | 2.63 | 2.23 | 0.41 |
| Naphthalene, 1-methyl- | 90-12-0 | 1.35 | 1.93 | 2.11 | 1.24 |
| Naphthalene | 91-20-3 | 5.42 | 11.07 | 9.06 | 6.49 |
| 1H-Pyrrole, 1-methyl- | 96-54-8 | 90.11 | 66.09 | 129.83 | 130.10 |
| Benzothiazole | 95-16-9 | 3.08 | 3.52 | 5.69 | 2.93 |
| Furan, 2-pentyl- | 3777-69-3 | 28.55 | 52.70 | 39.33 | 32.72 |
| Limonene | 138-86-3 | 5.08 | 7.49 | 12.05 | 4.11 |
